# Multiparametric MRI-based habitat and peritumoral radiomics integrating semi-automated segmentation for preoperative prediction of high-grade prostate cancer: a dual-center study

**DOI:** 10.3389/fonc.2026.1937448

**Published:** 2026-10-02

**Authors:** Danni Hua, Luyang Ma, Xiaodong Ji, Lixiang Huang, Yujiao Zhao, Wen Shen

**Affiliations:** 1 Department of Radiology, First Central Hospital of Tianjin Medical University, Tianjin, China; 2 Department of Radiology, Tianjin First Central Hospital, School of Medicine, Nankai University, Tianjin Institute of imaging medicine, Tianjin, China

**Keywords:** habitat imaging, high-grade prostate cancer, machine learning, multiparametric MRI, peritumoral radiomics, prostate cancer, radiomics

## Abstract

**Background:**

This study aimed to develop and validate an interpretable machine learning framework that combines semi-automated segmentation, intratumoral habitats derived from multiparametric MRI (mpMRI), and multiscale peritumoral radiomics to predict high-grade prostate cancer (HGPCa) before surgery.

**Methods:**

This retrospective dual-center study involved 274 patients, comprising 208 from Center 1 for development and 66 from Center 2 for external test. The clinical and imaging variables included patient age, serum PSA, fPSA, PSAD, maximum tumor diameter, PI-RADS scores, and anatomical zone distribution (PZ vs. TZ). The development cohort was randomly divided into a training cohort (n = 145) and an internal validation cohort (n = 63). Intratumoral habitats were delineated using semi-automated segmentation and unsupervised K-means clustering, while peritumoral radiomic features were extracted from concentric 1-, 3-, and 5-mm shells. Predictive classifiers were optimized through machine learning algorithms, particularly a multilayer perceptron (MLP) for the habitat signature. A combined clinical-radiomics nomogram was constructed, with SHapley Additive exPlanations (SHAP) employed to enhance model interpretability. Subgroup analyses were performed to assess the discriminative performance of the combined nomogram across the peripheral zone (PZ) and transition zone (TZ).

**Results:**

The combined nomogram exhibited superior diagnostic performance, achieving AUCs of 0.914, 0.903, and 0.797 in the training cohort, internal validation cohort, and external test cohort, respectively. The MLP-based habitat model showed good discriminative performance, with AUCs of 0.896, 0.897, and 0.782 across the training cohort, internal validation cohort, and external test cohort, respectively. Among the peritumoral regions, the 5-mm shell demonstrated the best discriminative performance. SHAP analysis identified Habitat 1 Shape Sphericity as the most influential feature. Subgroup analysis demonstrated the discriminatory ability of the combined nomogram in both the PZ (AUC: 0.796) and TZ (AUC: 0.753) subgroups.

**Conclusion:**

The integration of semi-automated segmentation-based habitat analysis and peritumoral radiomics provides a promising approach for preoperative risk stratification of prostate cancer.

## Introduction

Prostate cancer (PCa) continues to be a leading cause of cancer-related mortality among men globally (1). Patients classified within International Society of Urological Pathology (ISUP) grade groups 1–2 are generally considered suitable for active surveillance, which emphasizes quality of life by postponing invasive treatments. In contrast, HGPCa is linked to a markedly increased risk of biochemical recurrence, metastatic progression, and prostate cancer-specific mortality (2, 3). The clinical importance of this distinction is highlighted by significant prognostic disparities: high-grade tumors exhibit 5-year biochemical recurrence rates exceeding 45%, whereas lower-grade disease shows rates of less than 15% (4). The differentiation between low-to-intermediate-risk disease (ISUP grade groups 1–2) and high-grade prostate cancer (HGPCa, ISUP grade group ≥ 3) is essential for informed clinical decision-making.

Current preoperative evaluation relies heavily on the PI-RADS scoring system, which has become an established and widely adopted framework for risk stratification of prostate lesions. However, PI-RADS assessment remains primarily qualitative and may not fully capture the complex intratumoral heterogeneity associated with tumor aggressiveness (5). Although histopathological biopsy remains the diagnostic reference standard, its invasive nature underscores the need for reliable noninvasive tools to provide complementary information for personalized risk assessment (6).

Multiparametric MRI (mpMRI)-based radiomics offers a noninvasive approach to evaluate the aggressiveness of PCa (7). However, traditional radiomic techniques typically extract features from the entire tumor as a single region, thereby neglecting the spatial heterogeneity present within the lesion (8, 9). Additionally, these methods often concentrate solely on the intratumoral area, overlooking the peritumoral microenvironment—an adjacent region characterized by stromal remodeling and inflammation that has been implicated in tumor invasion (10). To address these shortcomings, recent studies have investigated intratumoral habitat analysis, employing unsupervised clustering of co-registered mpMRI maps (T2-weighted imaging, diffusion-weighted imaging, and apparent diffusion coefficient) to delineate microenvironmentally distinct subregions. Moreover, peritumoral radiomics captures features from concentric shells extending from the tumor boundary (11–13).

Recent advancements in deep learning have markedly improved prostate MRI segmentation by reducing manual effort and decreasing interobserver variability (14–16), thus establishing a standardized and objective basis for tumor analysis. Building on these complementary advances, we developed an interpretable machine learning framework that combines semi-automated segmentation, intratumoral habitat radiomics, and multiscale peritumoral radiomics for the preoperative prediction of HGPCa. Furthermore, we employed SHAP analysis to enhance model interpretability, thereby improving the interpretability of the model and potentially supporting clinical decision-making.

## Methods

### Study population

We consecutively enrolled patients with pathologically confirmed prostate cancer (PCa) who underwent multiparametric MRI (mpMRI) prior to diagnosis at Tianjin First Central Hospital (Center 1; January 2022–December 2025) and Tianjin Cancer Hospital (Center 2; January 2023–April 2026). This retrospective study was approved by the Science and Technology Ethics Committee of TFCH. The requirement for informed consent was waived because of the retrospective nature of the study.

A total of 274 patients met the eligibility criteria. The 208 patients from Center 1 constituted the development cohort, which was randomly stratified by pathological grade group (7:3 ratio) into a training cohort (n = 145) and an internal validation cohort (n = 63). The remaining 66 patients from Center 2 formed an independent external test cohort to assess model generalizability.

Inclusion criteria were as follows: (1) a definitive pathological diagnosis obtained through ultrasound-guided biopsy or radical prostatectomy; (2) no prior anti-tumor treatments, including surgery, radiotherapy, or chemotherapy; and (3) the availability of complete clinical and imaging data. Exclusion criteria included: (1) a history of other malignancies; (2) incomplete clinical or pathological records; and (3) severe image artifacts that precluded analysis. For patients with multifocal disease, the lesion exhibiting the highest ISUP grade group was selected for analysis. To account for scanner and acquisition variability, mpMRI examinations were performed using different scanner models and acquisition protocols across the two institutions (**Supplementary Table 1**). The overall study workflow is illustrated in **Figure 1**.

**Figure 1:**
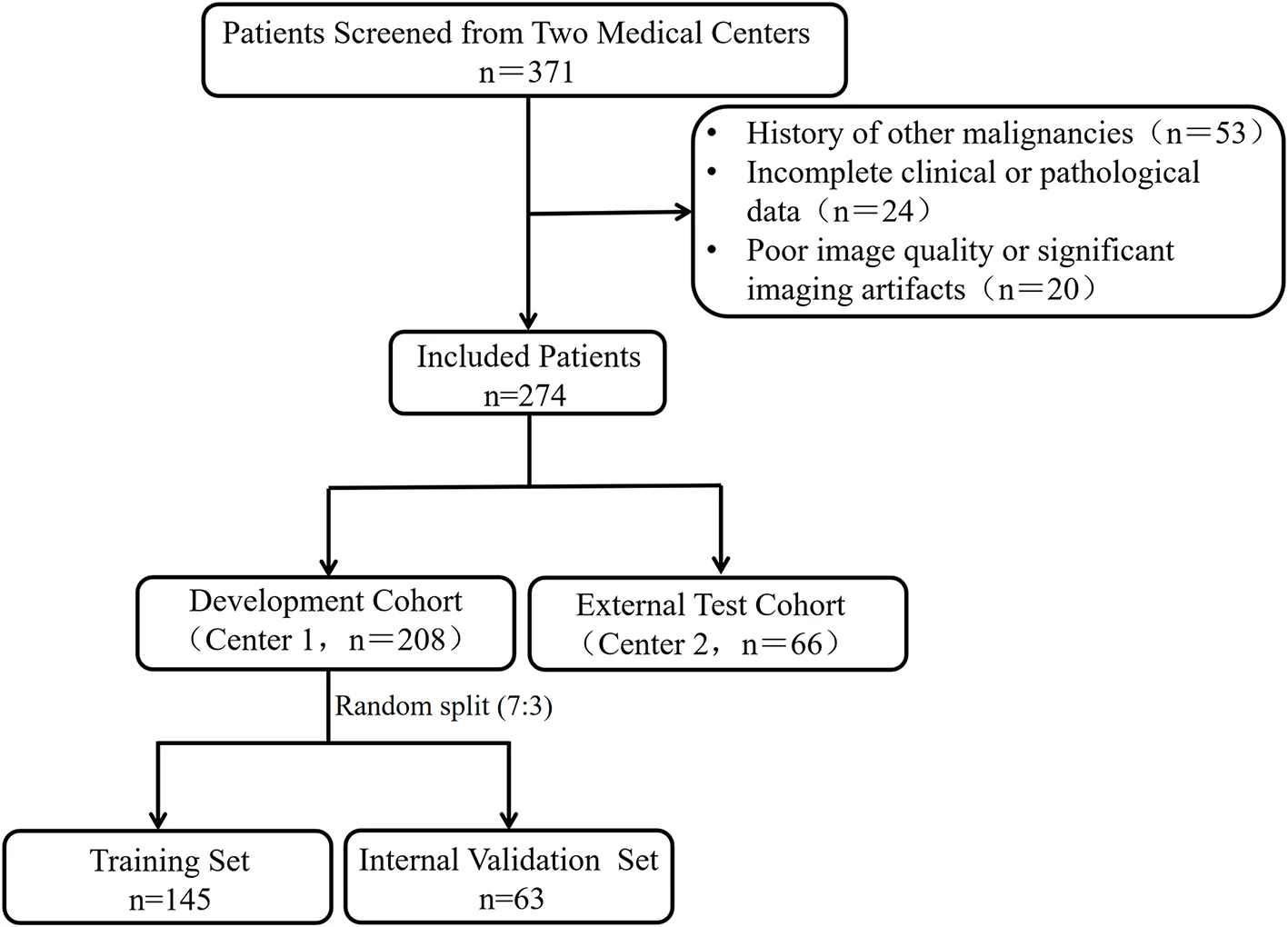
Study design and patient selection workflow. Flowchart illustrating patient enrollment, exclusion criteria, cohort allocation, and development of the multiparametric MRI-based predictive framework.

### Analytical framework overview

We established a multi-stage analytical framework that combines intratumoral habitat-based and multiscale peritumoral radiomics. The workflow included image preprocessing, semi-automated lesion segmentation, intratumoral habitat clustering, multiscale peritumoral feature extraction (utilizing 1-mm, 3-mm, and 5-mm concentric shells), machine learning model development, and ensemble performance assessment (**Figure 2**).

**Figure 2:**
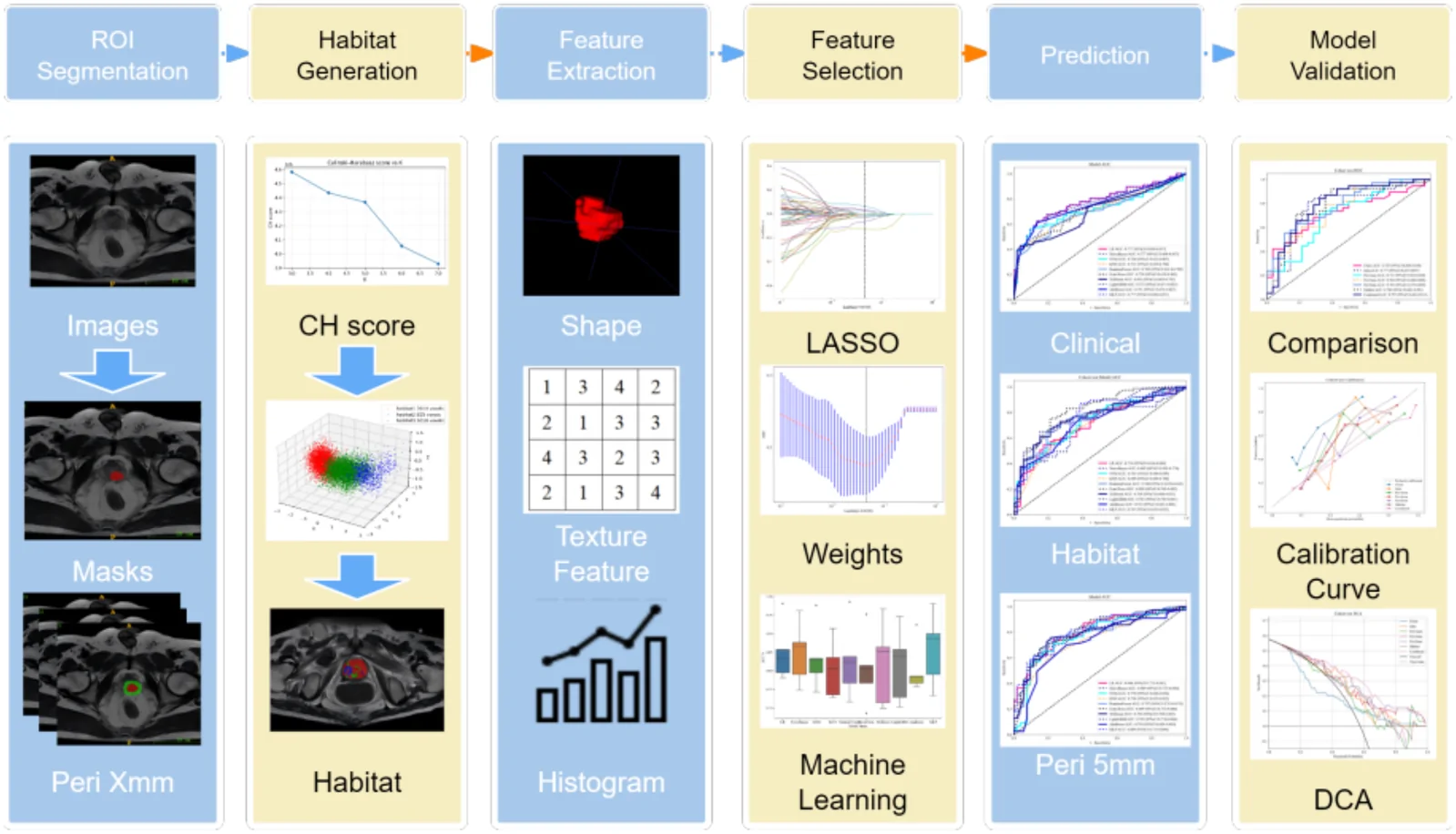
Overview of the proposed multiparametric MRI-based radiomics framework. Schematic illustration of automated segmentation, intratumoral habitat analysis, peritumoral radiomic feature extraction, machine learning modeling, and model interpretation using SHAP analysis.

### MRI acquisition and preprocessing

Multiparametric MRI (mpMRI) datasets, comprising axial T2-weighted imaging (T2WI), diffusion-weighted imaging (DWI, b = 1,000 s/mm²), and apparent diffusion coefficient (ADC) maps, were obtained from the Picture Archiving and Communication System (PACS) (**Figure 3A**). The preprocessing steps were conducted utilizing Python (v3.9.13) through a standardized pipeline: (1) N4 bias field correction; (2) rigid co-registration of DWI and ADC maps to the T2WI reference; (3) B-spline interpolation to achieve 1 × 1 × 1 mm³ isotropic voxels; and (4) Z-score intensity normalization.

**Figure 3:**
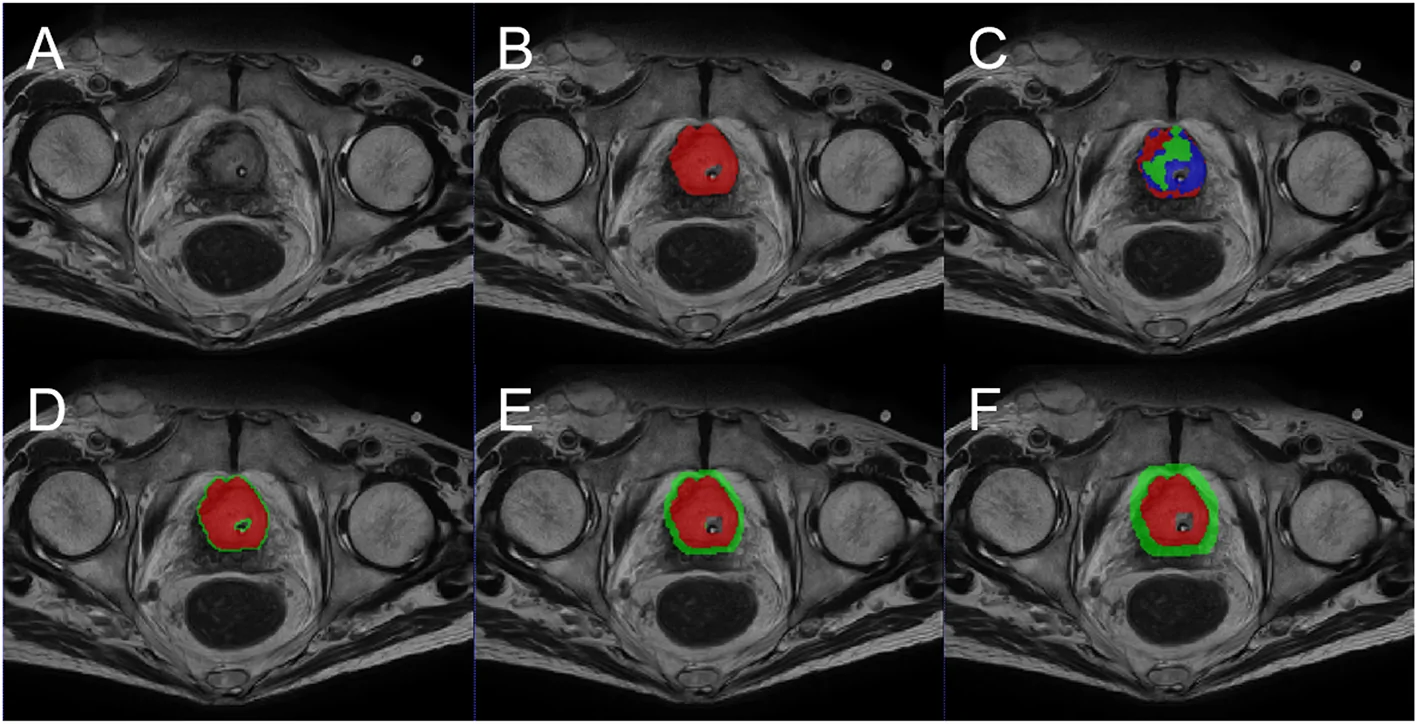
**(A)** Representative T2WI images. **(B)** Semi-automated tumor segmentation. **(C)** Intratumoral habitat generation using K-means clustering. **(D–F)** Peritumoral regions generated using 1-, 3-, and 5-mm expansions.

### Semi-automated segmentation and ROI definition

Primary tumor regions of interest (ROIs) were initially segmented using Nmstation, commercially available prostate MRI image analysis software developed through a collaborative research effort involving East China Normal University, Jiangsu Provincial People’s Hospital, and Shanghai Liwahe Intelligent Medical Technology Co., Ltd. The resulting ROIs were subsequently refined by two senior radiologists (8 and 10 years of experience, respectively) who were blinded to the histopathological findings. Necrotic, cystic, or hemorrhagic components were excluded from the ROIs (**Figure 3B**). Interobserver reliability was assessed in 20% of the cohort, resulting in an intraclass correlation coefficient (ICC) greater than 0.85. The accuracy of segmentation was validated using the Dice similarity coefficient, the 95% Hausdorff distance, and the average boundary distance.

### Habitat analysis

Intratumoral habitats were delineated using unsupervised K-means clustering, utilizing intensity features extracted from the co-registered mpMRI maps. The optimal number of clusters (K = 3) was determined based on the Calinski–Harabasz index and silhouette score (**Supplementary Figure 1**). The algorithm was executed for 100 iterations with randomized initializations (**Figure 3C**). K-nearest neighbors (KNN) imputation was utilized to address absent features in sparsely populated subregions, as needed.

### Peritumoral radiomics analysis

Peritumoral regions were delineated using OnekeyAI (v3.5.12) by isotropically expanding the whole-tumor ROI into concentric shells of 1-, 3-, and 5-mm (**Figures 3D–F**). Each shell represented the volumetric difference between the expanded and inner masks. Two senior radiologists reviewed these peritumoral rings and manually excluded adjacent non-prostatic structures, including the rectal wall and the bladder wall.

### Radiomic feature extraction and selection

Radiomic features were extracted in accordance with the Image Biomarker Standardization Initiative (IBSI) guidelines utilizing PyRadiomics. For each defined region comprising the entire tumor, three intratumoral habitats (H1–H3), and three peritumoral rings—107 features were extracted from T2WI, DWI, and ADC maps. These features included 14 3D shape features derived from the underlying masks, 18 first-order statistics, and 75 texture features obtained from gray-level co-occurrence matrices (GLCM), gray-level run-length matrices (GLRLM), gray-level size-zone matrices (GLSZM), gray-level dependence matrices (GLDM), and neighborhood gray-tone difference matrices (NGTDM) (**Supplementary Figure 2**). For habitat analysis, independent extraction across three subregions and three modalities resulted in 963 candidate features, which were subsequently integrated to construct the final habitat radiomics signature.

Feature selection was conducted within the training cohort through a four-step workflow. Initially, feature stability was assessed using the intraclass correlation coefficient (ICC) in a 20% replication subset, retaining features with an ICC of at least 0.85. Subsequently, after Z-score standardization, univariate analysis (independent-samples t-test) was performed to identify features associated with HGPCa (*P* < 0.05). This was followed by Pearson correlation analysis to remove redundant features (|r| > 0.80). Finally, least absolute shrinkage and selection operator (LASSO) regression with 10-fold cross-validation was employed to select optimal features based on the minimum cross-validated error (**Supplementary Figure 2**).

PI-RADS scores were independently assigned by two senior radiologists, who had 8 and 10 years of experience, respectively. Disagreements were resolved through consensus. The maximum tumor diameter and the peripheral zone/transition zone (PZ/TZ) ratio were calculated geometrically from the final segmentation masks and included in the clinical feature analysis.

### Model construction

Predictive signatures were developed for the clinical, intratumoral, peritumoral (1-, 3-, and 5-mm), and habitat profiles. Five machine learning classifiers—logistic regression (LR), Support Vector Machine (SVM), multilayer perceptron (MLP), K-nearest neighbors (KNN), and Extremely Randomized Trees (ExtraTrees)—were assessed using cross-validation within the training cohort. For each signature, the classifier demonstrating the highest mean performance was chosen: LR for the clinical and all peritumoral models; ExtraTrees for the intratumoral model; and MLP for the habitat model. For the habitat model, the MLP classifier comprised three hidden layers with 128, 64, and 32 neurons, respectively. The model was trained using the stochastic gradient descent (SGD) solver with a maximum of 300 iterations and a fixed random state of 0. Ultimately, a combined nomogram was constructed through multivariable logistic regression, integrating the clinical, habitat, and optimal peritumoral signatures to facilitate individualized HGPCa risk estimation.

### Statistical analysis

Statistical analyses were conducted using Python (v3.9.13) and R (v3.6.1). Continuous variables were assessed for distributional characteristics and compared among the three cohorts using one-way analysis of variance (ANOVA) or the Kruskal–Wallis test, as appropriate. Categorical variables were analyzed using the chi-square test or Fisher’s exact test. Model discrimination was assessed through receiver operating characteristic (ROC) analysis, with performance differences evaluated using the DeLong test. Clinical utility was examined via decision curve analysis (DCA), while interpretability was assessed using SHAP analysis.

## Results

### Patient characteristics

The development cohort was randomly divided into training and internal validation cohorts at a ratio of 7:3, while an independent cohort from another center was used as the external test cohort. Baseline clinical and imaging characteristics of the three cohorts are summarized in **Table 1**.

**Table 1:** Baseline characteristics of the study cohorts.

Variable	Training cohort (n = 145)	Internal validation cohort (n = 63)	External test cohort (n = 66)	Statistic	*P* value
Age (years)	73.43 ± 7.96	73.33 ± 7.73	69.05 ± 6.71	F = 8.167	<0.001
PSA (ng/mL)	35.04 ± 31.05	34.92 ± 34.51	23.11 ± 26.76	F = 2.980	0.052
fPSA (ng/mL)	6.97 ± 11.55	8.45 ± 13.88	3.87 ± 7.47	F = 2.131	0.121
f/t PSA	0.14 ± 0.09	0.15 ± 0.10	0.16 ± 0.09	F = 1.956	0.143
PSAD (ng/mL/cm³)	0.99 ± 1.32	0.94 ± 1.43	0.64 ± 1.22	F = 1.241	0.291
Maximum tumor diameter (mm)	43.35 ± 20.60	42.93 ± 20.25	27.89 ± 13.58	F = 16.221	<0.001
PZ/TZ ratio	0.58 ± 0.39	0.59 ± 0.41	0.75 ± 0.40	F = 4.154	0.017
Zone				χ² = 0.209	0.901
TZ	70 (48.3%)	31 (49.2%)	30 (45.5%)		
PZ	75 (51.7%)	32 (50.8%)	36 (54.5%)		
PI-RADS				χ² = 9.001	0.061
3	18 (12.4%)	10 (15.9%)	4 (6.1%)		
4	53 (36.6%)	20 (31.7%)	15 (22.7%)		
5	74 (51.0%)	33 (52.4%)	47 (71.2%)		
ISUP grade group				χ² = 15.272	0.054
1	38 (26.2%)	16 (25.4%)	17 (25.8%)		
2	34 (23.4%)	8 (12.7%)	11 (16.7%)		
3	30 (20.7%)	21 (33.3%)	9 (13.6%)		
4	14 (9.7%)	7 (11.1%)	15 (22.7%)		
5	29 (20.0%)	11 (17.5%)	14 (21.2%)		

Data are presented as mean ± standard deviation or number of patients, with percentages in parentheses. PSAD, prostate-specific antigen density; PZ, peripheral zone; TZ, transition zone.

### Validation of semi-automated segmentation

The automated segmentation generated by Nmstation exhibited strong concordance with manual delineation. The mean Dice similarity coefficient surpassed 0.85 across all MRI sequences, while the 95% Hausdorff distance (HD95) and average boundary distance (ABD) remained minimal, thereby indicating good geometric agreement between the automated and manual segmentations.

### Clinical feature selection

Univariable logistic regression analysis identified prostate-specific antigen (PSA), free prostate-specific antigen (fPSA), prostate-specific antigen density (PSAD), and maximum tumor diameter as significant predictors of high-grade prostate cancer (all *P* < 0.05). In the subsequent multivariable analysis, only maximum tumor diameter emerged as an independent predictor (OR = 1.019; 95% CI: 1.006–1.032; *P* = 0.013) and was employed to develop the clinical model (**Table 2**).

**Table 2:** Univariable and multivariable logistic regression analysis of clinical variables for high-grade prostate cancer.

Variable	Univariable		Multivariable	
	OR (95% CI)	*P* value	OR (95% CI)	*P* value
PZ/TZ ratio	0.724 (0.487–1.075)	0.179		
f/t PSA	0.987 (0.163–5.966)	0.991		
Age	1.001 (0.997–1.004)	0.785		
Zone	1.273 (0.868–1.866)	0.300		
Maximum tumor diameter	1.010 (1.004–1.016)	0.007	1.019 (1.006–1.032)	0.013*
PSA	1.020 (1.012–1.027)	<0.001	1.025 (0.997–1.053)	0.138
PI-RADS	1.036 (0.973–1.102)	0.350		
fPSA	1.096 (1.041–1.154)	0.003	1.039 (0.976–1.105)	0.315
PSAD	1.996 (1.454–2.740)	<0.001	1.504 (0.732–3.093)	0.351

CI, confidence interval; OR, odds ratio; PSA, prostate-specific antigen; PSAD, PSA density; PZ, peripheral zone; TZ, transition zone. *P < 0.05. Multivariable analysis included variables with P < 0.05 in univariable analysis.

### Radiomic signature and model construction

Following feature selection through ICC filtering, univariable analysis, and 10-fold cross-validated LASSO regression pipeline, the final number of features identified for each profile was as follows: 8 for the intratumoral signature, 12 for the habitat signature, and 8, 13, and 3 for the 1-, 3-, and 5-mm peritumoral regions, respectively (**Supplementary Figure 3**). Among the five machine learning classifiers assessed within the training cohort, the optimal models selected based on the highest mean performance were as follows: ExtraTrees for the intratumoral signature; MLP for the habitat signature; and LR for the 1-mm, 3-mm, and 5-mm peritumoral signatures.

### Diagnostic performance of predictive models

The combined model exhibited superior discriminative ability across all cohorts, achieving AUCs of 0.914, 0.903, and 0.797 in the training, internal validation, and external test cohorts, respectively (**Table 3**; **Figure 4**). Among the individual signatures, the habitat model maintained good discriminative performance, with AUCs of 0.896, 0.897, and 0.782 in the respective cohorts. Peritumoral optimization analysis showed that the 5-mm expansion achieved the highest AUC in the internal validation and external test cohorts, reaching an AUC of 0.785 in the external test cohort. Although several imaging-based models significantly outperformed the clinical model (DeLong test, P < 0.05), no significant difference was found between the combined and habitat models (P > 0.05) (**Supplementary Figure 4**). Finally, the nomogram (**Figure 5**) integrated the clinical, habitat, and 5-mm peritumoral signatures to facilitate individualized risk assessment, with the habitat signature contributing the largest range of points to the total score.

**Table 3:** Diagnostic performance of predictive models in the training, internal validation, and external test cohorts.

Signature	Accuracy	AUC	95% CI	Sensitivity	Specificity	PPV	NPV	Set
Clinic	0.779	0.806	0.733–0.878	0.658	0.903	0.873	0.722	Training
Intra	0.807	0.876	0.819–0.933	0.808	0.806	0.808	0.806	Training
Peri1-mm	0.786	0.852	0.790–0.914	0.795	0.778	0.784	0.789	Training
Peri3-mm	0.800	0.856	0.794–0.918	0.836	0.764	0.782	0.821	Training
Peri5-mm	0.800	0.844	0.778–0.910	0.712	0.889	0.867	0.753	Training
Habitat	0.862	0.896	0.843–0.951	0.822	0.903	0.896	0.833	Training
Combined	0.862	0.914	0.868–0.961	0.877	0.847	0.853	0.871	Training
Clinic	0.825	0.860	0.768–0.952	0.872	0.750	0.850	0.783	Internal Validation
Intra	0.810	0.866	0.779–0.953	0.744	0.917	0.935	0.687	Internal Validation
Peri1-mm	0.810	0.783	0.659–0.907	0.795	0.833	0.886	0.714	Internal Validation
Peri3-mm	0.794	0.856	0.763–0.948	0.692	0.958	0.964	0.657	Internal Validation
Peri5-mm	0.825	0.869	0.775–0.962	0.769	0.917	0.937	0.710	Internal Validation
Habitat	0.857	0.897	0.809–0.986	0.821	0.917	0.941	0.759	Internal Validation
Combined	0.889	0.903	0.817–0.989	0.872	0.917	0.943	0.815	Internal Validation
Clinic	0.697	0.727	0.605–0.850	0.684	0.714	0.765	0.625	External Test
Intra	0.788	0.777	0.657–0.897	0.816	0.750	0.816	0.750	External Test
Peri1-mm	0.697	0.711	0.583–0.838	0.711	0.679	0.750	0.633	External Test
Peri3-mm	0.727	0.764	0.648–0.880	0.789	0.643	0.750	0.692	External Test
Peri5-mm	0.712	0.785	0.675–0.895	0.632	0.821	0.828	0.622	External Test
Habitat	0.712	0.782	0.662–0.901	0.632	0.821	0.828	0.622	External Test
Combined	0.773	0.797	0.681–0.913	0.842	0.679	0.780	0.760	External Test

AUC, area under the receiver operating characteristic curve; CI, confidence interval; NPV, negative predictive value; PPV, positive predictive value; Int. Val, internal validation; Ext. Test, external test.

**Figure 4:**
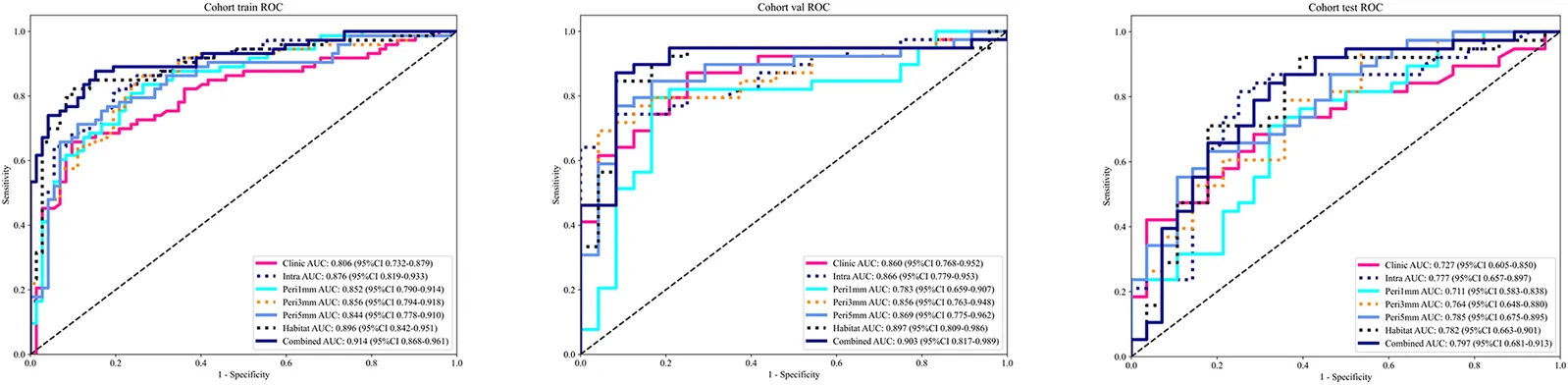
Receiver operating characteristic curves of different predictive models. ROC curves comparing the diagnostic performance of clinical, intratumoral radiomics, habitat radiomics, peritumoral radiomics, and combined models in the training cohort, internal validation cohort, and external test cohort.

**Figure 5:**
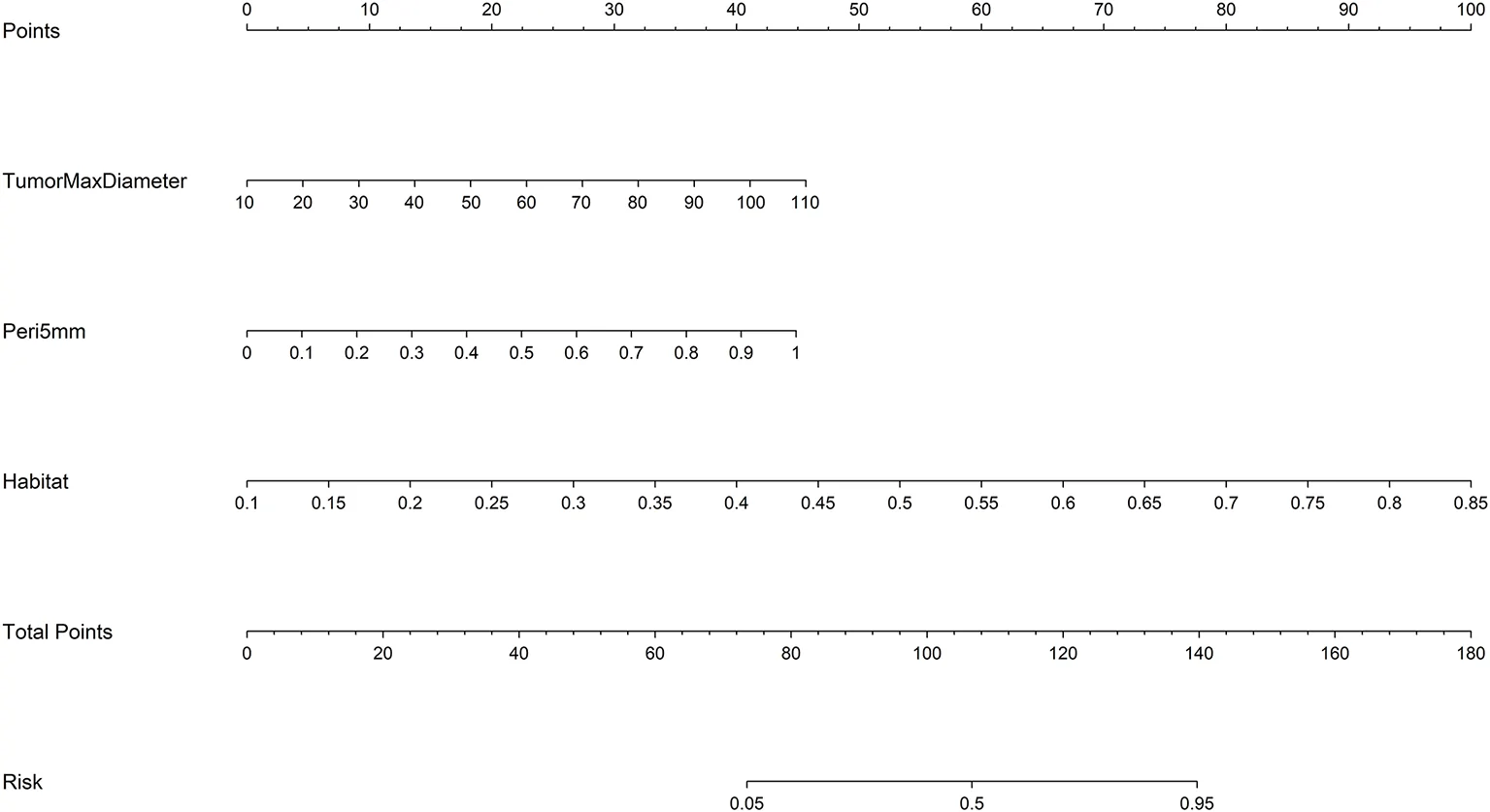
Construction and visualization of the combined prediction model. Nomogram developed by integrating clinical characteristics, habitat radiomics, and 5-mm peritumoral radiomic features for individualized prediction of high-grade prostate cancer.

To further assess model performance across anatomical zones, we conducted stratified subgroup analyses (**Table 4**). In the internal validation cohort, the combined model achieved AUCs of 0.865 and 0.929 in the PZ and TZ subgroups, respectively. In the external test cohort, the corresponding AUCs were 0.796 and 0.753, while the habitat model achieved AUCs of 0.757 and 0.747 in the PZ and TZ subgroups, respectively. Overall, both models demonstrated discriminatory ability across the two anatomical zones.

**Table 4:** Subgroup performance of the habitat and combined models in the internal validation and external test cohorts.

Set	Subgroup	Signature	AUC (95% CI)	Sensitivity	Specificity	F1 score	Accuracy
Internal Validation	PZ	Habitat	0.865 (0.694–0.992)	0.833	0.929	0.882	0.875
Internal Validation	PZ	Combined	0.865 (0.694–0.990)	0.833	0.929	0.882	0.875
Internal Validation	TZ	Habitat	0.924 (0.750–1.000)	0.810	0.900	0.872	0.839
Internal Validation	TZ	Combined	0.929 (0.773–1.000)	0.905	0.900	0.927	0.903
External Test	PZ	Habitat	0.757 (0.548–0.920)	0.625	0.895	0.714	0.771
External Test	PZ	Combined	0.796 (0.608–0.941)	0.750	0.684	0.706	0.714
External Test	TZ	Habitat	0.747 (0.504–0.951)	0.636	0.667	0.718	0.645

### Clinical utility evaluation

Calibration curves indicated a strong concordance between predicted and observed HGPCa risks across all datasets, with the combined model displaying the greatest calibration accuracy. Decision curve analysis (DCA) revealed that most radiomics models outperformed the clinical model in terms of net clinical benefit over a broad spectrum of thresholds. The combined model consistently yielded the highest net benefit, whereas the habitat signature retained significant clinical utility (**Figure 6**).

**Figure 6:**
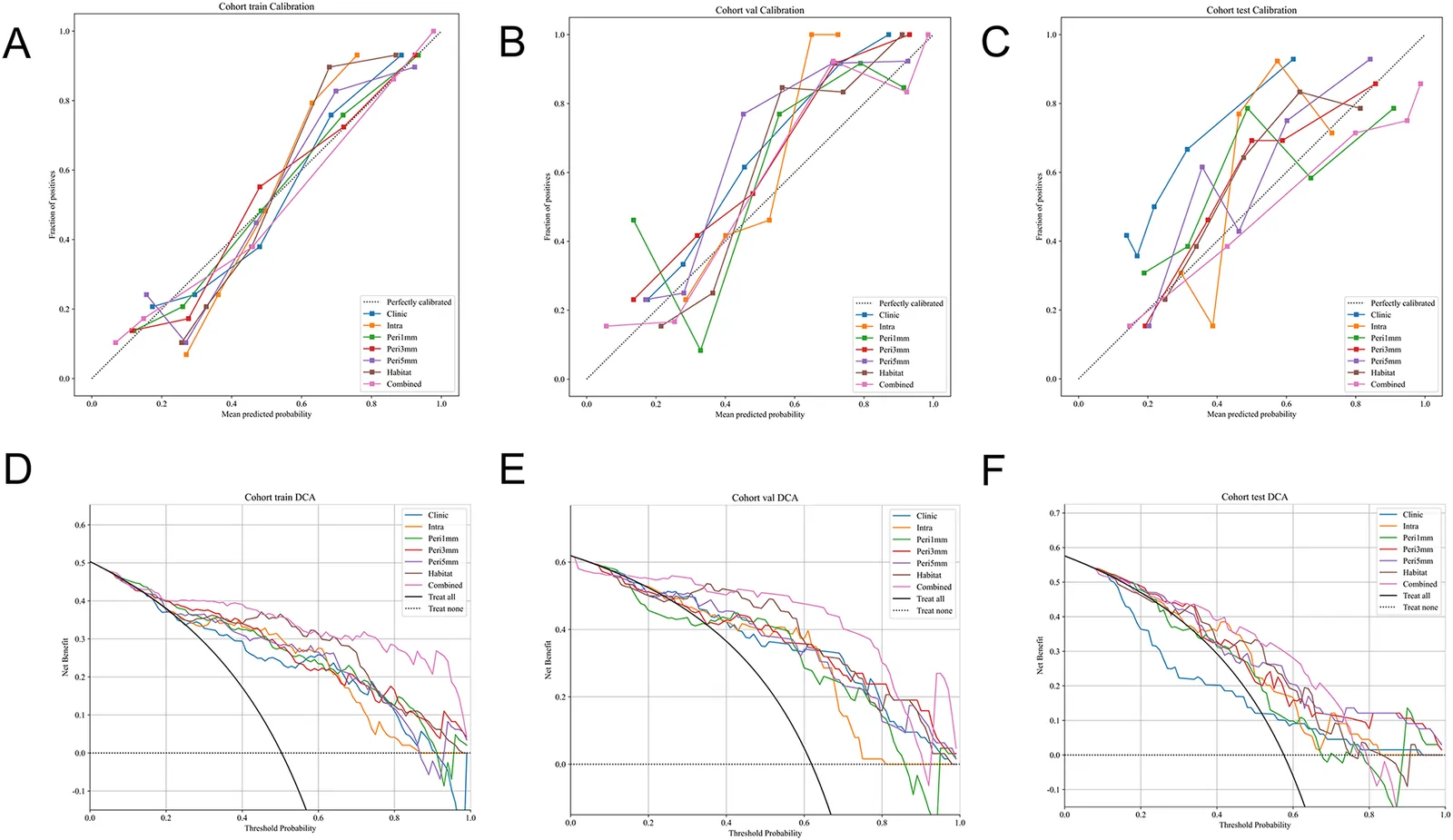
**(A–C)** Calibration curves for the training, internal validation, and external test cohorts, respectively. **(D–F)** Decision curve analysis for the training, internal validation, and external test cohorts, respectively.

### SHAP analysis

SHapley Additive exPlanations (SHAP) analysis was conducted to enhance the interpretability of the MLP-based habitat signature. The global SHAP summary plot ranked Shape Sphericity_Habitat 1_DWI, First-order Kurtosis_Habitat 2_DWI, First-order Skewness_Habitat 2_T2WI, and Shape Minor Axis Length_Habitat 2_DWI among the most influential features based on their mean absolute SHAP values. At the individual level, SHAP waterfall and force plots illustrated the specific contributions of features to specific model predictions, with Shape Sphericity_Habitat 1_DWI showing the largest contribution to the prediction of HGPCa (**Figure 7**).

**Figure 7:**
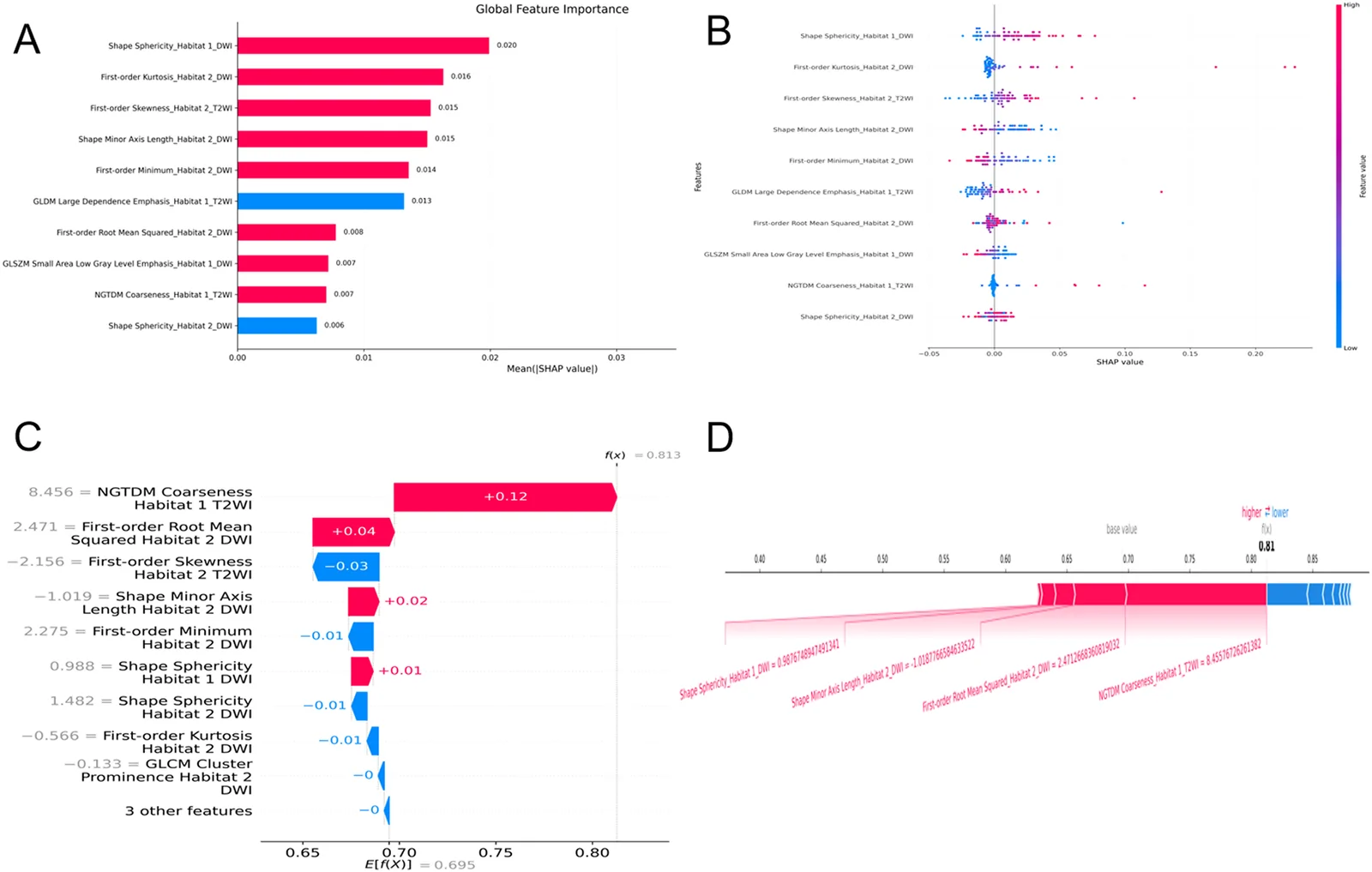
Interpretation of the combined model using SHAP analysis. **(A)** SHAP summary plot showing the contribution and direction of selected features. **(B)** SHAP waterfall plot illustrating feature-level contributions for an individual prediction case. **(C)** SHAP force plot demonstrating the cumulative impact of individual features on the predicted probability. **(D)** SHAP decision plot illustrating cumulative feature contributions.

## Discussion

In this study, we developed a nomogram integrating clinical information, semi-automated segmentation-based habitat analysis and multiscale peritumoral radiomics for the noninvasive preoperative prediction of high-grade prostate cancer (HGPCa). Validated across a dual-center cohort of 274 patients, the comprehensive framework exhibited good discriminative ability, with the combined model achieving AUCs of 0.914, 0.903, and 0.797 in the training, internal validation, and external test cohorts, respectively. Although the combined model incorporated complementary clinical information, its performance was not significantly superior to that of the habitat model, suggesting that the habitat model may represent a more parsimonious option for clinical implementation.

The foundation of this framework is a semi-automated ROI segmentation workflow utilizing Nmstation, which showed high geometric agreement (Dice >0.85) with manual delineation. The combined model maintained good discriminative performance in the external cohort, although the AUC decreased from 0.903 in the internal validation cohort to 0.797 in the external test cohort. This decrease may be partly related to inter-center differences in MRI scanners and acquisition parameters, as well as differences in baseline patient characteristics between the two cohorts, highlighting the need for multicenter validation and standardized MRI acquisition protocols.

In the clinical domain, maximum tumor diameter was identified as the sole independent predictor, whereas PSA, fPSA, and PSAD did not remain statistically significant. The independent association of maximum tumor diameter with HGPCa may reflect the relationship between tumor burden and aggressive disease, whereas PSA-related measures can be influenced by benign prostatic hyperplasia and prostatitis (17–20).

Habitat radiomics provided the dominant predictive information, while clinical and peritumoral signatures offered complementary information that enhanced the overall predictive performance of the model (21, 22). Subgroup analyses demonstrated that both the habitat and combined models retained discriminatory ability across the peripheral zone (PZ) and transition zone (TZ). In the external test cohort, the habitat model achieved a specificity of 0.895 in the PZ subgroup, suggesting relatively good specificity for identifying patients without HGPCa in this subgroup. However, the wide confidence intervals observed in the subgroup analyses indicate that these findings should be interpreted cautiously and require confirmation in larger cohorts.

SHAP analysis revealed that morphological and diffusion-weighted features in Habitat 1 and Habitat 2 serve as primary drivers of the habitat signature. Notably, *Shape Sphericity_Habitat 1_DWI* and *First-order Kurtosis_Habitat 2_DWI* were among the most influential features, potentially reflecting differences in intratumoral structural organization and signal-intensity heterogeneity (23, 24). Higher *Shape Sphericity_Habitat 1_DWI* values, reflecting more spherical and compact habitat geometry, were associated with a higher predicted probability of HGPCa. These findings improve the interpretability of the habitat model and may facilitate its future clinical translation.

Complementing the intratumoral analysis, our framework incorporated peritumoral radiomics and identified the 5-mm expansion shell as providing the best predictive performance. In contrast to the findings of Yuan et al. (25), which identified a 3-mm margin as optimal, this discrepancy may partly reflect differences in tumor size. Notably, tumors in our cohort were relatively larger than those reported by Yuan et al., and the broader 5-mm shell may therefore capture a wider range of peritumoral spatial heterogeneity (26, 27). Moreover, the combined model achieved a sensitivity of 0.909 in the TZ subgroup. This finding may be particularly relevant in the TZ, where benign prostatic hyperplasia can complicate MRI-based lesion assessment (28–31).

This study has several limitations. First, the retrospective design may introduce selection bias, although the independent external cohort provided an assessment of model generalizability. Second, a direct correlation with genomic mapping was not conducted, limiting the biological interpretability of our radiomic features. Third, MRI examinations were acquired using different scanners and acquisition protocols, and no feature-level harmonization was performed, which may affect radiomic feature stability and reproducibility. Future prospective multi-omics studies with larger and more diverse cohorts are warranted to enhance the biological interpretation and generalizability of our findings.

## Conclusion

In conclusion, we developed and externally validated a framework that integrates semi-automated segmentation, intratumoral habitats, and 5-mm peritumoral radiomics for noninvasive prediction of HGPCa. This model demonstrated good performance across the two centers, with SHAP interpretability. It may provide an interpretable decision-support tool for personalized preoperative risk stratification.

## Data Availability

The raw data supporting the conclusions of this article will be made available by the authors, without undue reservation.
