# Supplementary material for: Multiparametric MRI-based habitat and peritumoral radiomics integrating semi-automated segmentation for preoperative prediction of high-grade prostate cancer: a dual-center study
Source: Front Oncol. 2026 Oct 2;16:1937448. doi: 10.3389/fonc.2026.1937448 (PMC13671978; doi:10.3389/fonc.2026.1937448)
Supplement: Supplementary file 1 [file Table1.docx]

Supplementary Table 1 Detailed Multiparametric MRI Acquisition Parameters Across Centers and Scanners.

|  | Center 1 | | | | Center 2 | |
| --- | --- | --- | --- | --- | --- | --- |
| Scanner | SIEMENS Skyra 3T | | Ingenia 3T | | GE discovery 3T | |
| Sequence | T2 | DWI | T2 | DWI | T2 | DWI |
| TR (ms) | 5000 | 6000 | 5500 | 5700 | 6400 | 6300 |
| TE (ms) | 108 | 66 | 100 | 60 | 91 | 53 |
| Slice thickness (mm) | 3 | 3 | 3 | 3 | 3 | 3 |
| Intersection gap (mm) | 0.3 | 0.3 | 0.3 | 0.3 | 0.3 | 0.3 |
| FOV (mm2) | 260 | 260 | 260 | 260 | 280 | 280 |
| Matrix size | 320x320 | | 288x288 | | 382x320 | |

Supplementary Figure 1 Determination of the optimal number of intratumoral habitat clusters. (A) Calinski–Harabasz index and (B) silhouette score across different cluster numbers (K). The peak values consistently indicate that K=3 is the optimal number of subregions for the habitat analysis.

Supplementary Figure 2 Feature selection and LASSO regression performance. (A, B) Distribution and proportion of selected radiomic features. (C) Ten-fold cross-validation curve showing the mean squared error (MSE) as a function of the regularization parameter (λ). (D) LASSO coefficient profiles of the candidate features.

Supplementary Figure 3 Selected radiomic features across different models. (A) Intratumoral model. (B) Habitat model. (C–E) Peritumoral 1-mm, 3-mm, and 5-mm models, respectively.

Supplementary Figure 4 DeLong test results comparing differences in AUCs among predictive models in the training, internal validation, and external test sets. Heatmap visualization of pairwise comparisons is shown.
